# Modulation of glycation, inflammation, and detoxification pathways by D-Galactose through the RAGE–NF-κB–Nrf2 signaling axis in liver and kidney of Wistar rats

**DOI:** 10.1007/s13205-026-04988-5

**Published:** 2026-08-02

**Authors:** Nilima S. Bangar, Baishali Chakraborty, Rashmi S. Tupe

**Affiliations:** Symbiosis School of Biological Sciences (SSBS), Symbiosis International (Deemed University) (SIU), Lavale, Pune, Maharashtra 412115 India

**Keywords:** D-Galactose, Wistar rats, Inflammation, Glycation, Detoxification, Kidney, Liver

## Abstract

D-galactose (D-Gal) is used to induce aging-associated pathological complications; however, its dose-dependent effects on RAGE-NF-κB and Nrf2 pathways remain unexplored. We examine the effects of D-Gal (400, 500, and 600 mg/kg body weight) on glycation, inflammation, and detoxification parameters in the liver and kidney tissues of Wistar rats. Rats received subcutaneous D-Gal injections for 56 days, while the control rats received 0.9% saline. Blood samples were collected at 15-day intervals, and renal function was assessed using 24-h urine samples. After treatment, rats were sacrificed, liver and kidney tissues were harvested. D-Gal administration altered liver and kidney function by a dose-dependent increase in creatinine excretion upto 1.3-fold. Histological analysis revealed glomerular hypertrophy and hepatocellular injury. Additionally, an increase in GFR by 1.8-fold was observed (p < 0.05). D-Gal treatment showed a time-dependent increase in fructosamine (day 15), protein carbonyl content (day 30), and advanced glycation end products (AGEs) by day 56, accompanied by reduced free amino and thiol groups. Similar trends were observed in tissues, with D-Gal 500 showing the most pronounced effects (p < 0.001). Antioxidant and detoxification parameters exhibited time-dependent alterations, with changes in GSH levels and catalase activity in tissues (p < 0.001). D-Gal treatment significantly modulated RAGE and NF-κB expression at the gene and protein levels. D-Gal 600 induced a 7.5-fold increase in RAGE expression (p < 0.0001). NF-κB and Nrf2 expression were concentration-dependent, with changes at the protein level. Overall, D-Gal 500 exerted maximal effects via the RAGE-NF-κB-Nrf2 axis without significant cellular damage.

**Supplementary Information:**

The online version contains supplementary material available at 10.1007/s13205-026-04988-5.

## Introduction

The liver and kidneys are critical organs responsible for maintaining metabolic homeostasis, detoxifying substances, and excreting waste products. Disruption of their function is commonly associated with metabolic disorders characterized by oxidative stress, inflammation, and protein glycation (Francis et al. 2024; Devarbhavi et al. 2023). These pathological processes impair cellular integrity and organ function, leads to the progression of hepatic and renal diseases (Rad et al. 2024; Wang et al. 2024). Among the various factors, excessive exposure to reducing sugars plays an important role in metabolic dysfunction.

A reducing monosaccharide, D-galactose (D-Gal) is widely used to study aging and metabolic stress. Under physiological conditions, D-Gal is metabolized via the Leloir pathway; however, excess D-Gal leads to the accumulation of galactitol, which promotes osmotic imbalance and enhances the generation of reactive oxygen species (ROS) (Conte et al. 2021; Saafan et al. 2023; El-Far et al. 2024; Thakur et al. 2017). Elevated ROS levels accelerate non-enzymatic glycation reactions between reducing sugars and amino groups of protein, and through series of intermediate reactions finally generates the advanced glycation end products (AGEs) (Haase et al. 2024; Akagawa 2021; Tkemaladze 2024). These AGEs interact with the receptor for AGEs (RAGE), activates nuclear factor kappa B (NF-κB), leads to the production of pro-inflammatory cytokines and contributes to tissue inflammation and fibrosis (Yano et al. 2020; Son et al. 2017; Donate-Correa et al. 2021).

The transcription factor nuclear factor (erythroid-derived 2)-like 2 (Nrf2) is essential in regulating oxidative stress (Bellezza et al. 2018). When Nrf2 binds to the antioxidant response element in the nucleus, it increases the gene and protein expression of key enzymes, including heme oxygenase-1 (HO-1), NAD(P)H quinone oxidoreductase 1 (NQO1), and detoxification enzymes such as glyoxalase I and II (GLO I and II) (Kopacz et al. 2022). In contrast, persistent oxidative stress can inhibit Nrf2 activation, decreasing antioxidant capacity and impairing the detoxification of reactive intermediates like methylglyoxal. This imbalance between pro-oxidant and antioxidant mechanisms exacerbates cellular injury, contributing to liver and kidney dysfunction (Zeng et al. 2020; Saafan et al. 2023).

Earlier studies have demonstrated that D-Gal induces senescence (Shwe et al. 2018), oxidative stress (El-Far et al. 2024), and inflammation (Zeng et al. 2020). Most investigations have focused on specific molecular pathways independently. The integrated, dose-dependent effects of D-Gal on glycation, oxidative stress, antioxidant defense, and detoxification systems remain poorly understood. Also, the mechanistic relationship between the RAGE-NF-κB inflammatory axis and the Nrf2-mediated antioxidant response in liver and kidney tissues remains to be explored. Therefore, the present study hypothesizes that chronic D-Gal administration induces glycation and oxidative stress, activating the RAGE-NF-κB signaling pathway while simultaneously suppressing Nrf2-mediated antioxidant and detoxification responses. Here, we evaluated glycation, inflammation, antioxidant status, detoxification enzymes, and key signaling pathways across multiple doses of D-Gal (400, 500, and 600 mg/kg) at defined time points (0, 15, 30, 45, and 56 days). This study provides a detailed understanding of the time and dose-dependent molecular mechanisms underlying D-Gal-induced organ dysfunction.

## Materials and methods

### Chemicals

Various chemicals and reagents were obtained from registered suppliers for this study. MGO (Cat # M0252), 8-anilino-1-naphthalenesulfonic acid ammonium salt (ANS, Cat # A1028), S-lactoylglutathione (SLG, Cat # L7140), and benzoquinone (Cat # 12309) were purchased from Sigma Aldrich (St. Louis, USA). β-nicotinamide adenine dinucleotide phosphate (β-NADP +, Cat # 57647), reduced glutathione (GSH, Cat # 77285), and 5,5’-dithiobis (2-nitrobenzoic acid) (DTNB, Cat # 32363) were procured from Sisco Research Laboratories (SRL), India. D-galactose (Cat # GRM101), nitroblue tetrazolium chloride (NBT, Cat # RM 578), hydrogen peroxide (H₂O₂, Cat # Q18755), and radioimmunoprecipitation assay (RIPA) buffer (Cat # TCL 131) were obtained from Himedia, India. Biochemical assay kits, including those for albumin (Cat # 120200), creatinine (Cat # 120246), urea (Cat # 120241), serum glutamic oxaloacetic transaminase (SGOTCat # 120902), and serum glutamic pyruvate transaminase (SGPTCat # 120903), were purchased from Erba Mannheim (India). ELISA kits for IL-1β and TGF-β, and the respective antibodies, were obtained from Invitrogen (Waltham, MA, USA) and Santa Cruz Biotechnology, Inc. (Dallas, TX, USA). All remaining reagents and chemicals were of analytical grade. The primers for various genes were purchased from Bioserve Biotechnology Pvt., Ltd. (India).

### Animals and experimental design

Male albino Wistar rats (n = 24; 4 weeks old; 160–180 g) were obtained from Global Bioresearch Solutions Pvt. Ltd. Animals were housed under controlled conditions (25 ± 2 °C, 50–60% humidity, 12 h light/dark cycle) and had free access to food and water. The study was approved by the Institutional Animal Ethics Committee of Symbiosis School of Biological Sciences, Symbiosis International (Deemed University) (SSBS/IAEC/08-2023). After a one-week acclimatization period, rats were randomly assigned to four groups (n = 6 per group). D-Gal was dissolved in 0.9% saline and administered subcutaneously once daily at doses of 400, 500, and 600 mg/kg body weight to the respective groups for 56 days (Omidkhoda et al. 2020; Liu et al. 2017; Lew et al. 2020). The control group received an equivalent volume of 0.9% saline, and the injection volume was adjusted accordingly, with a maximum volume of 500 µL per animal (Turner et al. 2011). Blood samples (500 μL) were collected from the retro-orbital plexus into EDTA-coated vacutainers at days 0, 15, 30, 45, and 56 (Diehl et al. 2001). 24-h urine samples were collected using metabolic cages for renal function assessment. At the end of the experiment, animals were euthanized using ketamine-xylazine (1 mL/kg, i.p.) and perfused with 0.8% saline (Coman et al. 2022). Liver and kidney tissues were excised; one portion was fixed in 10% formalin for histological (H&E) and immunohistochemical analyses, while the remaining tissue was stored at − 80 °C for biochemical and molecular studies.

### Assessment of biochemical parameters and organ function

Plasma was separated from the collected blood samples by centrifugation at 2000 rpm for 10 min at 4 °C. Albumin, urea, creatinine, SGOT and SGPT levels in plasma and urine were measured using assay kits on an Erba Chem 7 analyzer (Transasia, Dubai, UAE). The somatic index for the liver and kidney was assessed by normalizing organ weight to total body weight, following the method outlined by Sertorio et al. (2019). Furthermore, glomerular filtration rate (GFR) was estimated by the creatinine clearance and blood urea nitrogen (BUN) clearance using the formula described by Pestel et al. (2007):

$$Creatinine\,clearance = \frac{{1000\,\, \times \,\,urine\,\,volume\,(mL) \times Creatinine\,in\,urine\,(\mu M/L)}}{{Creatinine\,in\,plasma\,(\mu M/L)}}$$

$$Bun\,clearance = \frac{{Urine\,volume\,(mL) \times BUN\,in\,urine\,(mM/L)}}{{BUN\,in\,plasma\,(mM/L)}}$$

$$GFR = \sqrt {Creatinine\,clearance \times \,BUN\,clearance}$$

Liver and kidney tissues were stained with H&E and examined under a bright-field microscope (Leica, Wetzlar, Germany). Histological changes, including glomerular volume (GV) and cytotoxicity, were quantified using ImageJ (v1.54d). GV was calculated from mean glomerular diameters, while cytotoxicity was assessed by the percentage of eosin-stained areas using the following formula (Khan et al. 2015).

$$GV = \left( {\beta /K} \right) \times \left( {GA} \right).^{{3/2}}$$

### Estimation of glycation markers and AGEs content

Liver and kidney tissues (500 mg each) were homogenized in RIPA buffer at a 1:8 (w/v) tissue-to-buffer ratio. After centrifugation of the homogenate at 12,000 rpm for 20 min at 4 °C (Eppendorf 5430R, Hamburg, Germany), the supernatant was collected and preserved at −20 °C for later analysis of glycation markers, antioxidant status, and detoxification enzymes. The Bradford method was used to determine the protein content in the tissue lysates (Kruger 2009).

#### Fructosamine content

Fructosamine levels in tissue lysates and plasma were estimated following the method reported by Baker et al. (1985) with minor modifications. The NBT reagent (400 µL, 0.75 mM) was dissolved in carbonate buffer (0.1 M, pH 10.35) and added to 20 µL of the respective samples, which were then incubated at 37 °C for 30 min. The absorbance of the incubated reaction mixture was measured at 530 nm using a Multiskan SkyHigh microplate spectrophotometer (Thermo Fisher Scientific, USA). Fructosamine content was measured using DMF as a standard (0–8 nM) and calculated from the calibration equation (Y = 0.0572x; R^2^ = 0.9669) and results were expressed as nM/mg protein.

#### Protein carbonyl content

Briefly, 50 μL of plasma or tissue sample was diluted with 200 μL of 1X PBS and added to 250 μL of DNPH (10 mM in 2.5 M HCl), then incubated at 37 °C for 1 h. Protein precipitation was performed with 1 mL of 10% TCA, and the mixture was centrifuged at 7,000 rpm for 10 min. The resulting pellet was washed three times with 1 mL of an ethanol:ethyl acetate (1:1 v/v) mixture and subsequently dissolved in 1 mL of 6 M urea. The absorbance of protein-hydrazone formation was measured at 365 nm, while the total protein concentration in the solution was determined at 280 nm (Uchida et al. 1998). The molar extinction coefficient of DNPH (ε at 365 nm = 21 mM⁻^1^·cm⁻^1^ was used for the estimation of the carbonyl concentration of proteins which were represented as nM carbonyl groups/mg of protein.

#### Free amino groups

The free amino groups in plasma or tissue lysates were estimated by the p-benzoquinone method (Aćimović et al. 2011). The 20 µL of benzoquinone (0.1 M in DMSO) was added to 50 µL of plasma or tissue lysate samples and 50 µL of phosphate-buffered saline. The samples were incubated at 37 °C for 30 min and measured at 480 nm. Quantification was performed using an alanine standard curve (0-100 nM), and concentrations were calculated using the calibration equation (Y = 0.0226x; R^2^ = 0.9983) and results were expressed as nM/mg protein.

#### Free thiol group

The free thiol group in the samples was determined using a previously reported method (Ellman 1959). To 125 µL of kidney tissue lysate, 375 µL of DTNB (0.5 mM) was added, and the mixture was incubated for 15 min at 25 °C. Further, the absorbance of the samples was read at 410 nm. The free thiol content was calculated using the molar extinction coefficient of DTNB (13.6 mM^−1^ cm^−1^) and expressed as nM of thiol/mg of protein.

#### AGEs content

To analyze the AGE content in tissue lysates, delipidation was performed (Soulis-Liparota et al. 1991). Briefly, 100 µL of tissue lysate was mixed with chloroform: methanol (2:1, v/v) and incubated overnight at 4 °C. The mixture was then centrifuged at 10,000 rpm for 30 min at 4 °C to separate the supernatant. The levels of different AGEs in the supernatant and plasma samples were analyzed using a fluorescence plate reader (BioTek Synergy H1, Vermont, USA) at their respective excitation/emission wavelengths: 335/400 nm for malondialdehyde, 370/440 nm for pentosidine, 485/530 nm for crossline, and 280/320 nm for tryptophan, as per Ahmed et al (2018) and Shamsi et al. (2020).

### Estimation of antioxidant markers

#### GSH content

The effect of D-Gal treatment on GSH content was analyzed using a previously reported method (Ellman 1959). Briefly, 250 μL of plasma or tissue lysate was mixed with 500 μL of a precipitating buffer (5% TCA in 1 mM EDTA) and then centrifuged at 2,000 rpm for 10 min. Next, 100 μL of the supernatant was diluted 2.5-fold with 0.1 M potassium phosphate buffer (pH 8.0), then 100 μL of DTNB (0.01%) was added, and the mixture was incubated at room temperature for 10 min. Afterwards, the absorbance was measured at 412 nm and results were expressed as nM/mg protein.

#### Catalase activity

Catalase activity was measured by the method reported by Aebi (1984). The 100 μL of plasma or tissue lysate was meticulously mixed with 560 μL of 50 mM phosphate buffer (pH 7). The reaction was initiated by the addition of 333 μL of H_2_O_2_ (30 mM in phosphate buffer). The absorbance of the samples was measured at 240 nm at 0 and 60 s. Enzyme activity was determined using the molar extinction coefficient of H_2_O_2_ (43.6 mM-1 cm-1 at 240 nm).

### Estimation of detoxification markers

#### GLO I activity

GLO I activity in the plasma or tissue lysates was analyzed by the method described elsewhere (Arai et al. 2014). To prepare the reaction mixture, 500 µL of sodium phosphate buffer (100 mM, pH 6.6) was mixed with 100 µL each of GSH (20 mM) and MGO (20 mM), followed by the addition of 280 µL of distilled water. The mixture was incubated at 37 °C for 10 min. The enzyme activity was started by adding 20 µL of plasma or tissue lysates, and absorbance at 240 nm was measured over five minutes at one-minute intervals. Enzyme activity was expressed in U/mg protein.

#### GLO II activity

The GLO II enzyme activity was analyzed spectrophotometrically with a slight modification to the reported method (Arai et al. 2014). For this purpose, 380 µL of distilled water, 500 µL of Tris–HCl (100 mM, pH 7.4), and 100 µL of SLG solution (3 mM) were added, and the reaction was initiated by the addition of 20 µL of plasma or tissue lysate. The absorbance was measured at 240 nm with one-minute intervals over five minutes. Enzyme activity was represented as U/mg protein.

#### Aldose reductase (AR) Activity

A spectrophotometric method was employed to evaluate AR activity (Jung et al. 2008). The reaction was initiated by mixing 20 µL of the plasma or tissue lysate with the mixture of 50 mM potassium phosphate buffer (480 μL), 0.2 M lithium sulfate (100 μL), 10 mM glyceraldehyde (200 μL), and 66 mM NADPH (200 μL). The absorbance was measured at 340 nm over three minutes. AR activity was then represented as U/mg protein.

### Real-time quantitative polymerase chain reaction

Total RNA was extracted from 100 mg of hepatic and renal tissues using 1 mL of TRIzol reagent according to the method described by Rio et al. (2010). The purity of the extracted RNA samples was assessed using a µDrop Plate (Thermo Fisher Scientific, Waltham, Massachusetts, USA). RNA samples exhibiting an A260/A280 ratio of 2.0 were used for cDNA synthesis with the iScript cDNA Synthesis Kit according to the manufacturer's instructions. The gene expression of was evaluated with the primer sequences are provided in Supplementary Table S1 using the CFX96 RT-PCR system (Bio-Rad, California, USA). The PCR protocol for the genes included an initial denaturation at 95 °C for 3 min, followed by 40 cycles of denaturation at 95 °C for 10 s, annealing at 60 °C for 30 s, and extension at 72 °C for 10 s. The relative expression level of the target gene compared to the internal control (GAPDH) was calculated using the 2^-^^ΔΔCt^ method. Results were presented as fold changes relative to the control group or untreated cells, as reported by Sharma et al. 2018.

### Detection of IL-1β and TGF-β1 in the plasma by ELISA

ELISA kits specific for IL-1β and TGF-β1 were used to measure plasma levels of these cytokines in the treated animals. The assay was conducted following the manufacturer's instructions. The calibration curves of IL-1β (312.5–2500 pg/mL; Y = 6E-05x-0.0327; R^2^ = 0.962) and TGF-β1 (156.25–2500 ng/mL; Y = 0.0002x – 0.0957; R^2^ = 0.98) were generated and used to calculate the cytokine concentration.

### Elucidation of protein expression using the Western blot technique

Western blotting was performed to evaluate the effect of D-Gal on protein expression (Adeshara et al. 2018). Initially, 25 µg of protein from tissue lysates after mixing with 4X loading dye separated by 12% SDS-PAGE. The separated protein was then transferred to nitrocellulose membranes (95 V, 120 min). To prevent non-specific binding of the antibody, blocking was done with 5% BSA and 2 h incubation at room temperature on a shaker. After incubation, the membrane was washed once with TBST for 10 min, and primary antibodies at different dilutions-Nrf2 (1:2500), RAGE, NF-κB, and β-actin (1:1000) were added and kept at 4 °C overnight. Next, the membrane was washed and incubated with HRP-conjugated secondary antibodies (anti-rabbit, 1:10,000; anti-mouse, 1:1000). Detection was performed using a chemiluminescence kit (SYNGENE G:BOX, UK), and band intensity was quantified by densitometry with ImageJ software (v1.44p, NIH, USA).

### Immunostaining of Nrf2 and NF-κB in renal tissue by immunohistochemical (IHC) analysis

Tissue sections were deparaffinized, antigen-retrieved, and blocked with 3% H₂O₂ and 100 μL goat serum. After incubation with Nrf2 and NF-κB antibodies (1:500), PolyExcel reagents and DAB were applied. Slides were counterstained, dehydrated, and imaged using a bright-field microscope (Leica DM2500 microscope, Wetzlar, Germany) (Liu et al. 2022). The blinded slide evaluation method was used to grade immunostaining (0–5) based on intensity and distribution.

### Statistical analysis

One-way ANOVA was used to assess statistical significance, followed by Tukey’s post-hoc test in GraphPad Prism (version 8.0.1). Two types of statistical comparisons were made- (1): comparison with the control group indicated by *; (2) comparison with D-Gal 600 group indicated by $. The significance was indicated as follows: ^*, $^ (p < 0.05); ^**, $$^ (p < 0.01); ^***, $$$^ (p < 0.001); and ^****,^ (p < 0.0001). Relationship between different parameters was evaluated by Pearson’s correlation analysis.

## Results

### D-Gal induced renal and hepatic dysfunction: histological and biochemical assessment

**Fig. 1:**
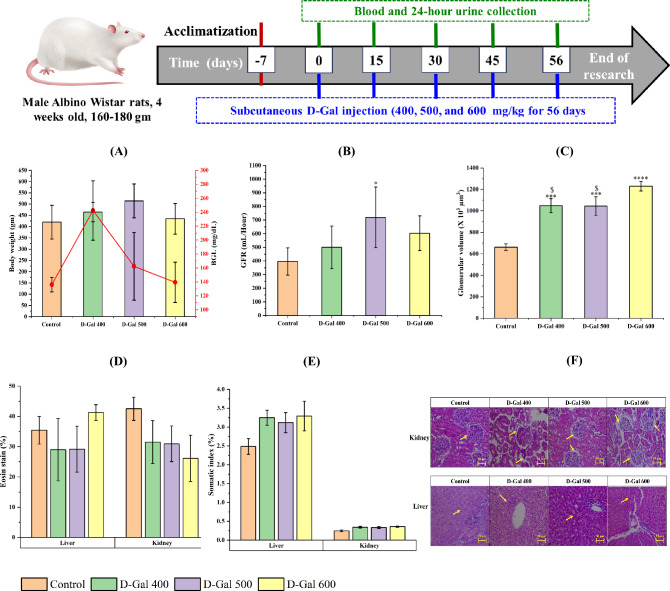
Effect of chronic D-galactose (D-Gal) administration on physiological, renal, and histopathological parameters in male Wistar rats. Experimental animals received subcutaneous D-Gal injections (400, 500, and 600 mg/kg body weight) for 56 days. **A** Changes in body weight and blood glucose level (BGL) in control and D-Gal-treated groups. **B** Glomerular filtration rate (GFR) is altered following D-Gal administration. **C** Quantitative analysis of glomerular volume indicates glomerular hypertrophy in treated groups. **D** Percentage eosin-stained area in liver and kidney tissues representing tissue injury and histochemical alterations. **E** Hepatic and renal somatic index (HSI and RSI), calculated as organ weight relative to body weight. **F** Representative hematoxylin and eosin (H&E)-stained Sects. (400X) of kidney and liver tissues showing histopathological alterations in D-Gal-treated animals, including glomerular enlargement, tubular degeneration, inflammatory infiltration, and disruption of normal tissue architecture (yellow arrows). Data are expressed as mean ± SD (n = 6). (*) Comparison with the control group, ($) comparison with D-Gal 600. Significance was indicated as follows: *, $ (p < 0.05); **, $$ (p < 0.01); ***, $$$ (p < 0.001); and ****, $$$$ (p < 0.0001)

Chronic D-Gal administration resulted in time-dependent changes in biochemical markers of kidney and liver function (Table 1). The urea level was increased on day 15 in the D-Gal 500 group, indicating early renal stress. Across all D-Gal groups, SGOT and SGPT levels were elevated, particularly at day 56, resulting in progressive hepatic injury. Albumin levels declined mildly at later time points in the D-Gal 400 and 600. Additionally, 24-h urinary analysis (Table 2) revealed increased excretion of creatinine and urea, particularly in the D-Gal 500 and 600 groups. As shown in Fig. 1A, body weight remained relatively unchanged among all groups, although a slight reduction was observed in the D-Gal 600 group. Interestingly, BGL showed a significant elevation in the D-Gal 400 group, followed by a marked decline at the 500 and 600 groups. Renal function analysis (Fig. 1B) revealed a statistically significant increase in GFR in the D-Gal 500 (p < 0.05) compared to control, while no significant changes were noted in the D-Gal 400 and 600 groups. Notably, the D-Gal 600 group showed a progressive increase in glomerular volume (p < 0.0001), followed by the D-Gal 500 (p < 0.01) and D-Gal 400 (p < 0.01) groups (Fig. 1C), compared to the control. The eosin-stained area percentage was increased in both liver and kidney tissues. The prominent changes were observed in the D-Gal 500 group (Fig. 1D). Furthermore, the somatic index (Fig. 1E) showed a significant increase in liver weight relative to body weight across all D-Gal-treated groups. However, kidney somatic indices remained statistically unchanged. D-Gal-treated rats displayed enlarged glomeruli, mesangial expansion, and tubular dilation (indicated by yellow arrows), particularly in the D-Gal 500 and 600 groups. Liver sections revealed hepatocyte ballooning, sinusoidal dilatation, and mild inflammatory infiltration, which were more evident at higher doses, especially in the D-Gal 600 group (Fig. 1F). D-Gal administration induced dose-dependent morphological alterations in liver and kidney tissues, including discoloration and surface irregularities (Supplementary Fig. 1).

**Table 1:** Effect of D-Gal treatment on the biochemical parameters of plasma samples at different time periods

Experimental groups	Samples→	Plasma				
	Parameters→	Albumin (g/dL)	Creatinine (mg/dL)	Urea (mg/dL)	SGOT (U/L)	SGPT (U/L)
	Days↓					
Control	0^th^ day	3.19 ± 0.29	1.51 ± 0.62	20.43 ± 8.95	35.36 ± 10.0	21.21 ± 2.49
D-Gal 400		3.32 ± 0.18	1.63 ± 0.39	10.87 ± 2.79	42.87 ± 17.64	22.39 ± 3.67
D-Gal 500		3.62 ± 0.36	1.04 ± 0.16	18.67 ± 5.67	42.78 ± 8.96	18.56 ± 6.03
D-Gal 600		3.68 ± 0.32	2.10 ± 0.97	17.93 ± 7.22	51.27 ± 12.4	8.84 ± 2.4^***^
Control	15^th^ day	3.84 ± 0.25	2.01 ± 0.20	5.16 ± 3.97	55.16 ± 7.43	28.47 ± 3.62
D-Gal 400		3.86 ± 0.26	1.76 ± 0.25	20.21 ± 9.19	56.57 ± 2.49	37.13 ± 9.99
D-Gal 500		3.94 ± 0.10	1.55 ± 0.20	39.06 ± 20.95	54.81 ± 1.28	36.24 ± 1.25
D-Gal 600		3.78 ± 0.22	1.49 ± 0.51	29.28 ± 20.29	52.95 ± 1.02	42.43 ± 11^***^
Control	30^th^ day	3.95 ± 0.34	1.62 ± 0.07	8.26 ± 2.54	34.24 ± 5.97	23.45 ± 2.34
D-Gal 400		3.86 ± 0.26	1.65 ± 0.17	30.21 ± 8.27^***^	55.69 ± 4.67^***^	38.49 ± 4.84
D-Gal 500		3.94 ± 0.10	1.62 ± 0.16	45.26 ± 10.14^***^	54.81 ± 0^***^	30.64 ± 3.67
D-Gal 600		3.78 ± 0.22	1.47 ± 0.33	35.24 ± 5.21^***^	60.7 ± 4.08^***^	32.71 ± 6.25
Control	45^th^ day	3 ± 0.37	1.17 ± 0.25	9.54 ± 1.24	48.32 ± 1.01	36.54 ± 2.70
D-Gal 400		2.46 ± 0.23	1.11 ± 0.35	35.21 ± 4.28^***^	46.85 ± 4.44	36.68 ± 4.17
D-Gal 500		3.08 ± 1.27	0.96 ± 0.15	42.13 ± 5.04^***^	45.97 ± 9.84	36.24 ± 3.75
D-Gal 600		3.03 ± 0.26	0.88 ± 0.19	39.23 ± 2.54^***^	40.66 ± 4.99	28.64 ± 4.03
Control	56^th^ day	2.66 ± 0.59	1.28 ± 0.53	5.49 ± 0.59	60.11 ± 17.62	46.55 ± 5.40
D-Gal 400		2.66 ± 0.50	1.66 ± 0.31	9.11 ± 1.47	72.84 ± 16.97	52.74 ± 24.55
D-Gal 500		3.21 ± 0.28	1.62 ± 0.86	11.23 ± 0.60	50.83 ± 6.01	56.57 ± 19.88
D-Gal 600		2.83 ± 0.75	1.5 ± 0.43	9.78 ± 1.53	58.34 ± 17.81	54.80 ± 5.30

Data are presented as mean ± SD. Two types of comparisons were made: (*) comparison with the control group, ($) comparison with D-Gal 600

Significance was indicated as follows: ^*, $^ (p < 0.05); ^**, $$^ (p < 0.01); ^***, $$$^ (p < 0.001); and ^****,^ (p < 0.0001)

Abbreviations: *D-Gal*: D-galactose

**Table 2:** Effect of D-Gal treatment on the biochemical parameters of a 24-h collected urine sample of rat

Experimental groups	Creatinine (mg/dL)	Urea (mg/dL)
Control	19.75 ± 6.60	0.72 ± 0.15
D-Gal 400	22.95 ± 2.99	0.90 ± 0.19
D-Gal 500	25.48 ± 5.53	1.12 ± 0.16
D-Gal 600	27.49 ± 2.70	0.92 ± 0.20

Data are presented as mean ± SD. Two types of comparisons were made: (*) comparison with the control group, ($) comparison with D-Gal 600

Significance was indicated as follows: ^*, $^ (p < 0.05); ^**, $$^ (p < 0.01); ^***, $$$^ (p < 0.001); and ^****,^ (p < 0.0001)

Abbreviations: *D-Gal:* D-galactose

### Alteration of glycation markers and functional groups in liver and kidney tissues

**Fig. 2:**
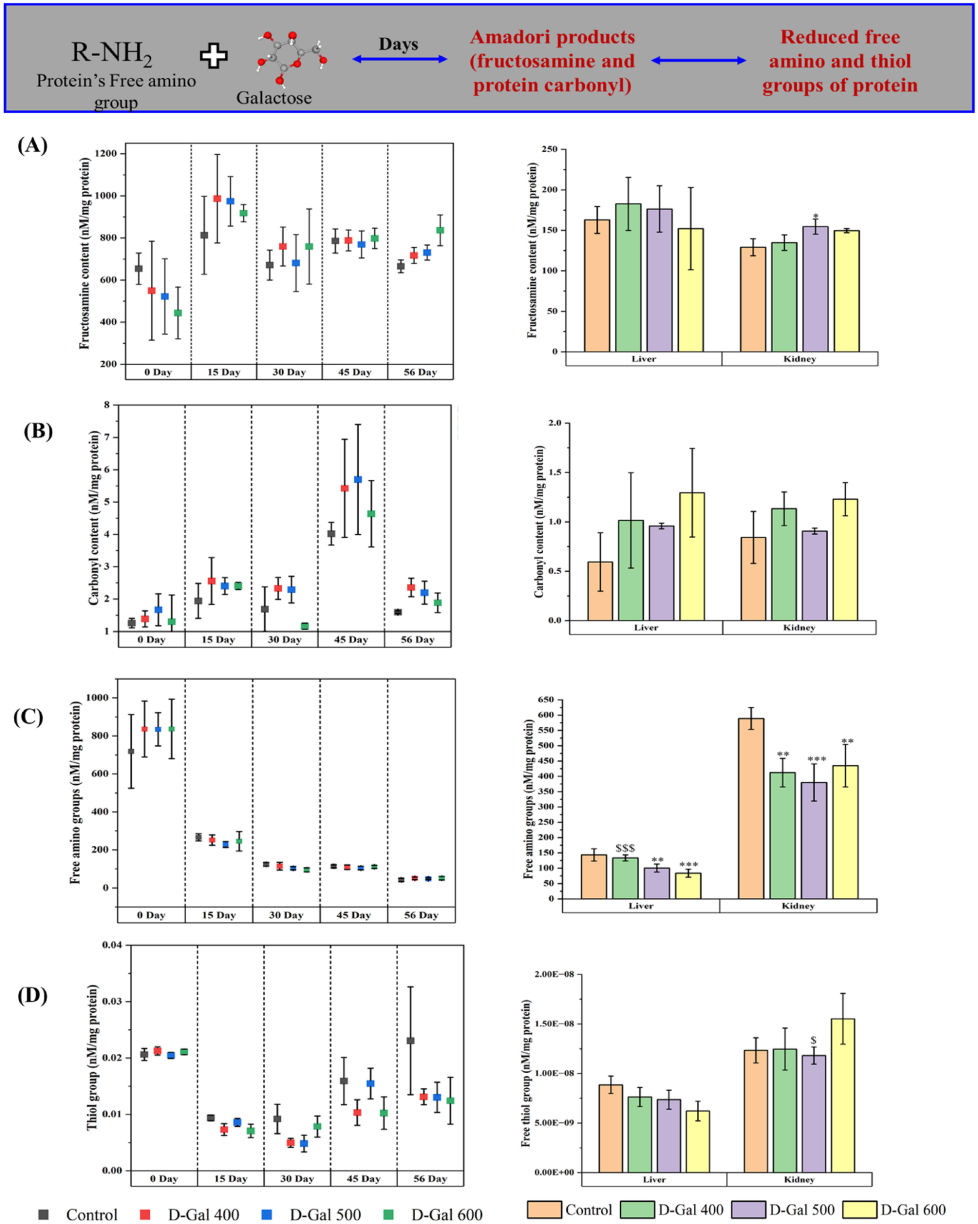
Effect of chronic D-galactose (D-Gal) administration on glycation-associated biochemical markers in plasma, liver, and kidney tissues. The schematic illustrates the interaction of D-Gal with free amino groups on proteins, leading to the formation of Amadori products (fructosamine and protein carbonyls) and a reduction in free amino and thiol groups. **A** Fructosamine content was measured in plasma at different experimental time points (0, 15, 30, 45, and 56 days) and in liver and kidney tissue lysates. **B** Protein carbonyl content measured in plasma and tissue lysates. **C** Quantification of free amino groups in plasma, liver, and kidney tissues. **D** Estimation of free thiol groups in plasma and tissue lysates. The left panels represent time-dependent changes in plasma biochemical parameters, while the right panels show endpoint analysis of liver and kidney tissue lysates. Data are expressed as mean ± SD (n = 6). (*) Comparison with the control group, ($) comparison with D-Gal 600. Significance was indicated as follows: *, $ (p < 0.05); **, $$ (p < 0.01); ***, $$$ (p < 0.001); and ****, $$$$ (p < 0.0001)

Chronic D-Gal exposure induced time- and tissue-dependent alterations in protein glycation and oxidative stress markers (Fig. 2). Plasma fructosamine showed a transient increase at day 15, with no sustained changes thereafter; however, the highest levels were observed in the D-Gal 600 group at day 56 (Fig. 2A). Tissue fructosamine levels showed only mild, non-significant changes. Protein carbonyl content peaked at day 45 and declined by day 56 in plasma, with the highest levels in the D-Gal 500 group (Fig. 2B). In contrast, carbonyl content was consistently elevated in liver and kidney tissues, with maximal levels in the D-Gal 600 group. Free amino groups declined after day 15 in plasma and remained stable thereafter (Fig. 2C). In liver tissue, a significant dose-dependent reduction was observed (D-Gal 400 < 500 < 600; p < 0.01–0.001), while the kidney showed a similar but non-significant trend. Plasma thiol content decreased progressively across all D-Gal groups (Fig. 2D), accompanied by reduced levels in liver tissue. In contrast, kidney tissue in the D-Gal 600 group retained relatively higher thiol levels, suggesting tissue-specific antioxidant compensation.

### AGE accumulation and oxidative stress in plasma, liver, and kidney

**Fig. 3:**
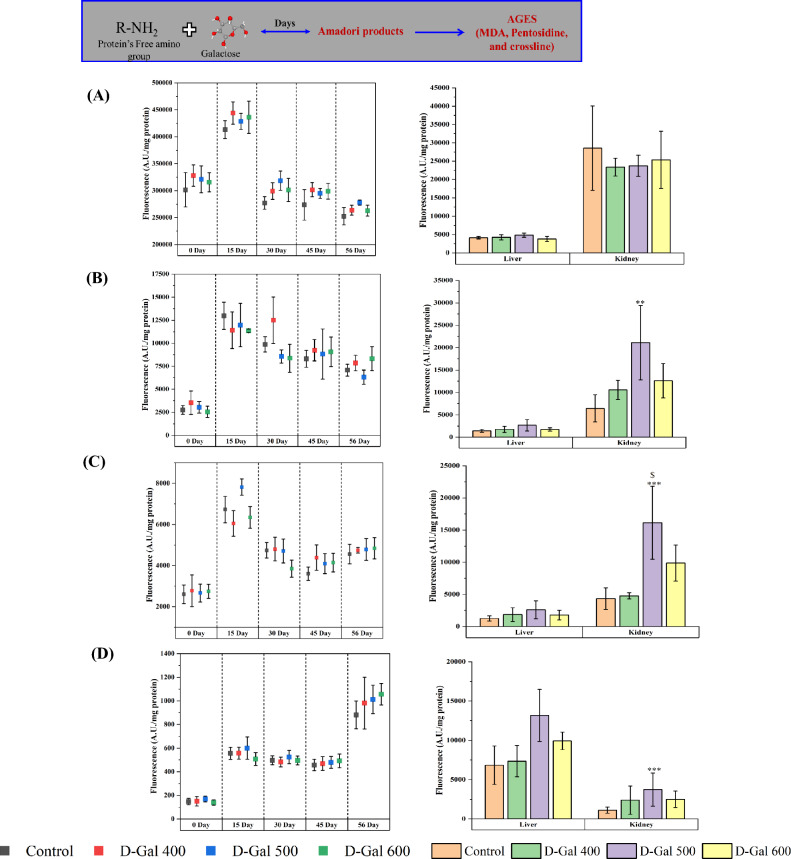
Effect of chronic D-galactose (D-Gal) administration on advanced glycation end products (AGEs) in plasma, liver, and kidney tissues. Male Wistar rats were administered subcutaneous injections of D-Gal (400, 500, and 600 mg/kg body weight) for 56 days to evaluate the progression of advanced glycation and oxidative stress. The schematic illustrates the glycation pathway, in which the interaction of D-Gal leads to the generation of AGEs, including malondialdehyde (MDA), pentosidine, and crosslinking products. **A** Tryptophan fluorescence was measured in plasma at different experimental time points (0, 15, 30, 45, and 56 days) and in liver and kidney tissue lysates. **B** Malondialdehyde (MDA) levels were determined in plasma and tissue lysates as an indicator of lipid peroxidation and oxidative stress induced by D-Gal treatment. **C** Pentosidine fluorescence quantified in plasma, liver, and kidney tissues. **D** Crossline fluorescence measured in plasma and tissue lysates to assess AGE-mediated protein modification and crosslink formation. The left panels represent time-dependent changes in plasma AGE-related markers during the experimental period, while the right panels show endpoint analysis in liver and kidney tissue lysates. Data are expressed as mean ± SD (n = 6). (*) Comparison with the control group, ($) comparison with D-Gal 600. Significance was indicated as follows: *, $ (p < 0.05); **, $$ (p < 0.01); ***, $$$ (p < 0.001); and ****, $$$$ (p < 0.0001)

Plasma tryptophan fluorescence showed a transient increase at day 15, followed by a decline at later time points (Fig. 3A), with the greatest reduction observed in the D-Gal 600 group by day 56. Tissue analysis revealed no significant change in liver tryptophan content, while a non-significant decrease was observed in the kidney. A similar temporal trend was observed for MDA-derived fluorescence in plasma (Fig. 3B), with maximal levels in the D-Gal 600 group at day 56. Liver MDA levels remained largely unchanged, except for a significant increase in the D-Gal 500 group (p < 0.01), while in the kidney, no significant changes were observed. Pentosidine levels in plasma increased at day 15 across all D-Gal groups and declined thereafter (Fig. 3C), with higher levels in the D-Gal 500 and 600 groups. In liver tissue, a modest increase was observed, particularly in the D-Gal 500 group. In kidney tissue, MDA levels were highest in the D-Gal 500 group, followed by D-Gal 600 and 400. Crossline, a late-stage AGE, showed maximal plasma levels at day 56 in the D-Gal 500 and 600 groups (Fig. 3D). Similarly, liver and kidney tissues exhibited the highest crossline content in the D-Gal 500 group, with a significant increase in the kidney (p < 0.001).

### Antioxidant depletion in plasma, liver, and kidney tissues

**Fig. 4:**
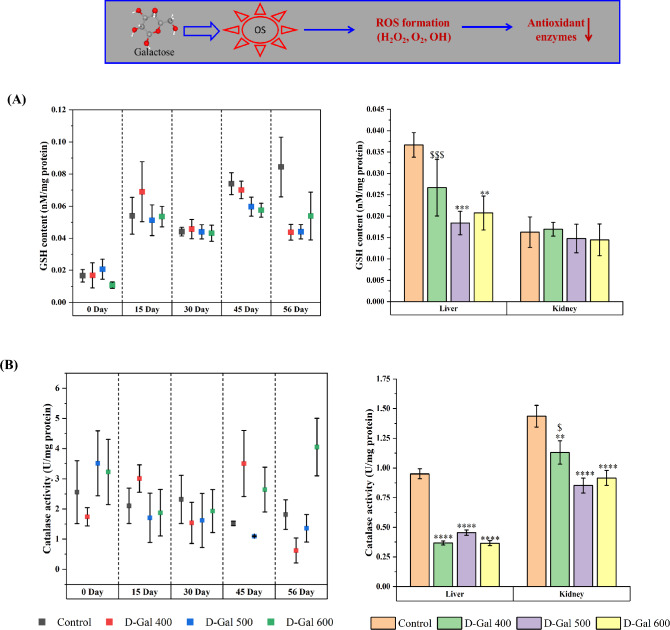
Effect of chronic D-galactose (D-Gal) administration on antioxidant defense status in plasma, liver, and kidney tissues. Male Wistar rats were administered subcutaneous injections of D-Gal (400, 500, and 600 mg/kg body weight) for 56 days. The schematic illustrates D-Gal-mediated oxidative stress (OS), which generates reactive oxygen species (ROS), including hydrogen peroxide (H₂O₂), superoxide radicals (O₂• −), and hydroxyl radicals (•OH), thereby impairing antioxidant defense mechanisms. **A** Reduced glutathione (GSH) content was measured in plasma at different experimental time points (0, 15, 30, 45, and 56 days) and in liver and kidney tissue lysates. **B** Catalase activity was measured in plasma and tissue lysates as an indicator of enzymatic antioxidant defense against ROS-mediated oxidative damage. The left panels represent time-dependent changes in plasma antioxidant parameters during the experimental period, whereas the right panels show endpoint analysis of liver and kidney tissue lysates. Data are expressed as mean ± SD (n = 6). (*) Comparison with the control group, ($) comparison with D-Gal 600. Significance was indicated as follows: *, $ (p < 0.05); **, $$ (p < 0.01); ***, $$$ (p < 0.001); and ****, $$$$ (p < 0.0001)

Plasma GSH levels declined over time, with marked reductions in the D-Gal 500 and 600 groups by day 56 (Fig. 4A). In liver tissue, GSH content was significantly decreased in all D-Gal groups (p < 0.01–0.001), whereas no significant changes were observed in the kidney. Plasma catalase activity showed temporal fluctuations but was overall reduced in the D-Gal 500 group by day 56 (Fig. 4B). Tissue-specific comparisons revealed a dramatic decline in liver catalase activity in all D-Gal groups. Kidney catalase activity was also significantly reduced in D-Gal 400 (p < 0.01), 500 (p < 0.0001), and 600 (p < 0.0001) groups.

### Detoxification enzyme activity in the plasma, liver, and kidney

**Fig. 5:**
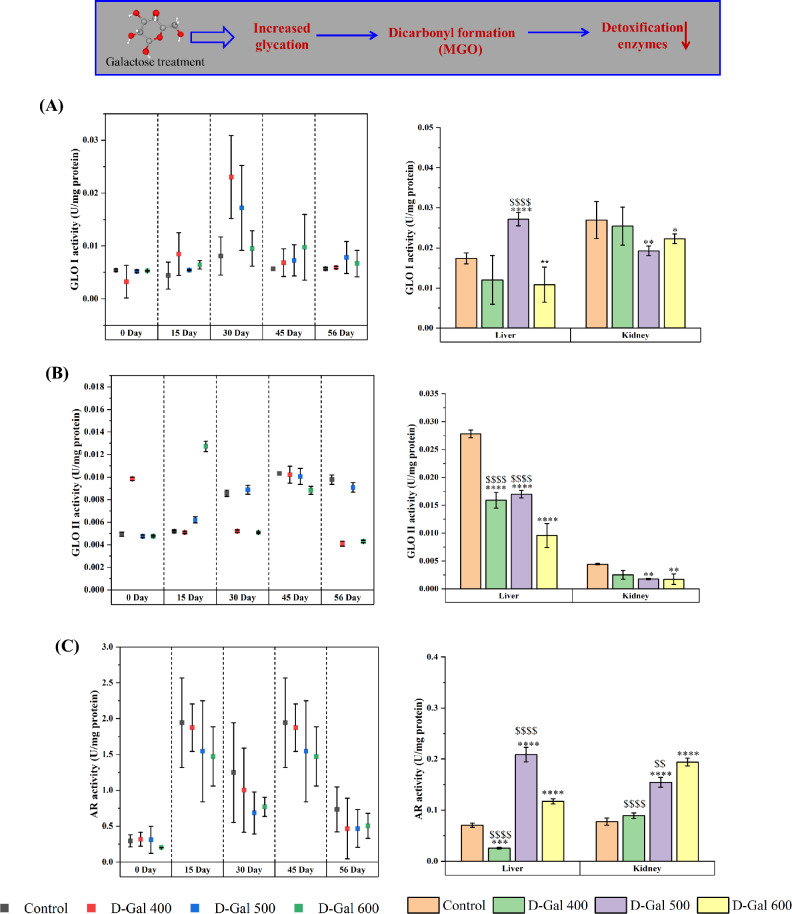
Effect of chronic D-galactose (D-Gal) administration on detoxification enzyme activities in plasma, liver, and kidney tissues. Male Wistar rats were administered subcutaneous injections of D-Gal (400, 500, and 600 mg/kg body weight) for 56 days. The schematic illustrates D-Gal-induced glycation, leading to increased formation of dicarbonyl compounds, particularly methylglyoxal (MGO), and impaired detoxification enzyme activity. **A** Glyoxalase I (GLO I) activity was measured in plasma at different experimental time points (0, 15, 30, 45, and 56 days) and in liver and kidney tissue lysates. **B** Glyoxalase II (GLO II) activity in plasma and tissue lysates. **C** Aldose reductase (AR) activity measured in plasma, liver, and kidney tissues Data are expressed as mean ± SD (n = 6). (*) Comparison with the control group, ($) comparison with D-Gal 600. Significance was indicated as follows: *, $ (p < 0.05); **, $$ (p < 0.01); ***, $$$ (p < 0.001); and ****, $$$$ (p < 0.0001)

In plasma (Fig. 5A), GLO-I activity remained largely unchanged over time. In contrast, liver GLO-I activity was significantly reduced in the D-Gal 400 and 600 groups, while an unexpected increase was observed in the D-Gal 500 group (p < 0.0001). No significant changes in kidney GLO-I activity were detected. Plasma GLO-II activity increased over time in the D-Gal 500 group (Fig. 5B), whereas liver GLO-II activity was reduced in a dose-independent manner, with a significant decline in the D-Gal 600 group (p < 0.001). Plasma AR activity showed an initial increase followed by a marked decline by day 56, particularly in the D-Gal 500 group (Fig. 5C). In liver tissue, AR activity was elevated in the D-Gal 500 (p < 0.01) and 600 (p < 0.0001) groups. In contrast, kidney AR activity increased in a dose-dependent manner (D-Gal 400 < 500 < 600).

### RAGE–NF-κB–Nrf2 axis and inflammatory responses in liver and kidney

**Fig. 6:**
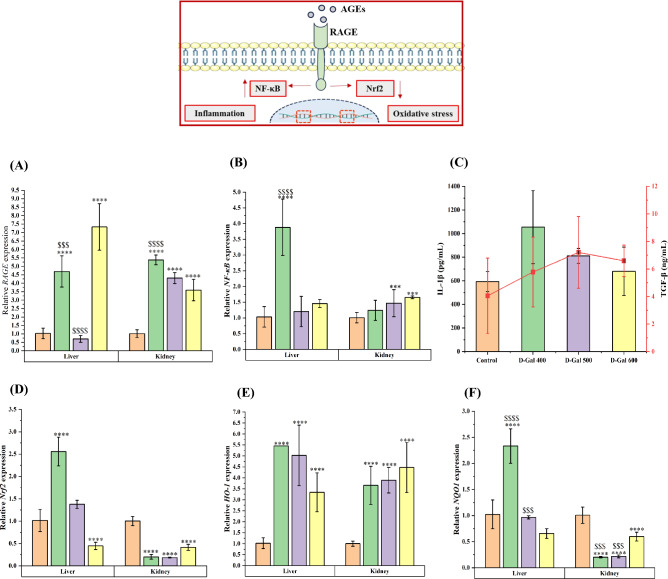
Effect of chronic D-galactose (D-Gal) administration on inflammatory and antioxidant signaling pathways in liver and kidney tissues and cytokine levels in plasma. Male Wistar rats were administered subcutaneous injections of D-Gal (400, 500, and 600 mg/kg body weight) for 56 days. **A** Relative mRNA expression of receptor for advanced glycation end products (RAGE) in liver and kidney tissues. **B** Relative mRNA expression of nuclear factor kappa B (NF-κB). **C** Plasma levels of interleukin-1 beta (IL-1β) and transforming growth factor beta (TGF-β). The bar graph represents IL-1β levels, while the red line indicates TGF-β concentration. **D** Relative mRNA expression of nuclear factor erythroid 2-related factor 2 (Nrf2). **E** Relative mRNA expression of heme oxygenase-1 (HO-1). **F** Relative mRNA expression of NAD(P)H quinone dehydrogenase 1 (NQO1). The results demonstrate that chronic D-Gal exposure modulates AGE–RAGE-mediated inflammatory signaling while altering antioxidant defense responses in liver and kidney tissues. Data are expressed as mean ± SD (n = 6). (*) Comparison with the control group, ($) comparison with D-Gal 600. Significance was indicated as follows: *, $ (p < 0.05); **, $$ (p < 0.01); ***, $$$ (p < 0.001); and ****, $$$$ (p < 0.0001)

D-Gal treatment in rats significantly affected RAGE gene expression. As shown in Fig. 6A, in liver tissue, the D-Gal 400 group exhibited a significant increase in RAGE expression (p < 0.0001) compared to the control, followed by the D-Gal 600 and 500 groups. A similar trend was observed in kidney tissue, where the D-Gal 400 group showed the highest RAGE expression, followed by the D-Gal 500 and 600 groups. Furthermore, as depicted in Fig. 6B, increased RAGE expression was associated with the upregulation of NF-κB gene expression. In liver tissue lysates, the D-Gal 400 group showed the highest NF-κB expression (p < 0.0001) compared to the other D-Gal groups. However, in kidney tissue lysates, a dose-dependent increase in NF-κB expression was observed (D-Gal 400 < D-Gal 500 < D-Gal 600). Plasma levels of IL-1β and TGF-β (Fig. 6C) further supported this trend, with elevated IL-1β levels in the D-Gal 400 group and a progressive increase in TGF-β levels, indicating systemic inflammation and fibrotic signaling. Importantly, the D-Gal 500 and D-Gal 600 groups (p < 0.0001) showed significant downregulation of Nrf2 expression in both tissues, suggesting impaired antioxidant defense (Fig. 6D). Furthermore, downstream antioxidant genes HO-1 and NQO1 (Fig. 6E and F) followed a similar pattern, with markedly reduced expression in the D-Gal 500 and 600 groups compared to controls, particularly in kidney tissues.

### Protein and immunohistochemical expression of NF-κB and Nrf2 in liver and kidney tissues

**Fig. 7:**
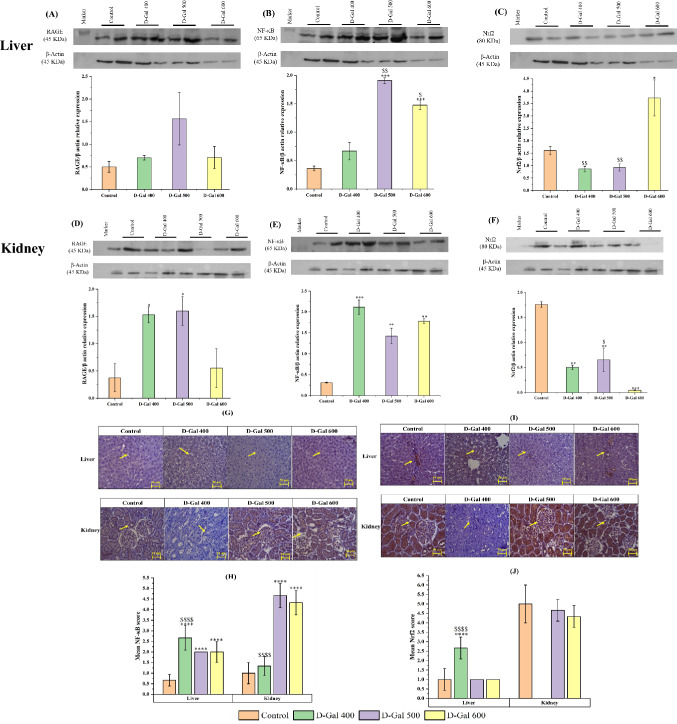
Effect of chronic D-galactose (D-Gal) administration on protein expression and immunohistochemical localization of RAGE, NF-κB, and Nrf2 in liver and kidney tissues. Male Wistar rats were administered subcutaneous injections of D-Gal (400, 500, and 600 mg/kg body weight) for 56 days. Western blot images and densitometric analyses demonstrate alterations in the expression of inflammatory and antioxidant signaling proteins in liver and kidney tissues. β-Actin was used as the internal loading control. **A**–**C** Protein expression in liver tissue showing relative expression of (**A**) receptor for advanced glycation end products (RAGE), **B** nuclear factor kappa B (NF-κB), and **C** nuclear factor erythroid 2-related factor 2 (Nrf2). **D**–**F** Protein expression analysis in kidney tissue showing relative expression of **D** RAGE, **E** NF-κB, and **F** Nrf2. G Representative immunohistochemical staining of NF-κB in liver and kidney tissues. **H** Semi-quantitative mean NF-κB immunohistochemical score in liver and kidney tissues. **I** Representative immunohistochemical staining of Nrf2 in liver and kidney tissues. **J** Semi-quantitative mean Nrf2 immunohistochemical score in liver and kidney tissues. Yellow arrows indicate immunopositive DAB-stained cells or regions. The results demonstrate that chronic D-Gal exposure modulates AGE–RAGE-associated inflammatory signaling through increased NF-κB activation and altered Nrf2-mediated antioxidant responses in hepatic and renal tissues. Data are expressed as mean ± SD (n = 6). (*) Comparison with the control group, ($) comparison with D-Gal 600. Significance was indicated as follows: *, $ (p < 0.05); **, $$ (p < 0.01); ***, $$$ (p < 0.001); and ****, $$$$ (p < 0.0001)

Western blot analysis showed that in liver tissue, RAGE and NF-κB expression levels were significantly elevated in the D-Gal 500 and 600 groups (Fig. 7A and 7B). Whereas Nrf2 expression was significantly downregulated in these groups, indicating suppression of antioxidant defense mechanisms (Fig. 7C). Similar trends were observed in kidney tissue, where D-Gal 500 and 600 treatments significantly upregulated RAGE and NF-κB expression (Fig. 7D and E) and significantly reduced Nrf2 expression (Fig. 7F), indicating a shift toward oxidative and inflammatory signaling. IHC analysis revealed elevated NF-κB staining intensity in hepatocytes and renal tubular cells of the D-Gal–treated groups (Fig. 7G). Quantitatively, NF-κB immunoreactivity was significantly upregulated in both the liver and kidney at doses of 500 and 600 mg/kg. Quantification revealed a significant reduction in mean Nrf2 scores in the liver at 500 and 600 mg/kg (Fig. 7H). Interestingly, in kidney tissues, Nrf2 staining remained relatively stable across groups (Fig. 7I and J).

### Comparative biochemical and correlation analysis of D-gal treatment

**Fig. 8:**
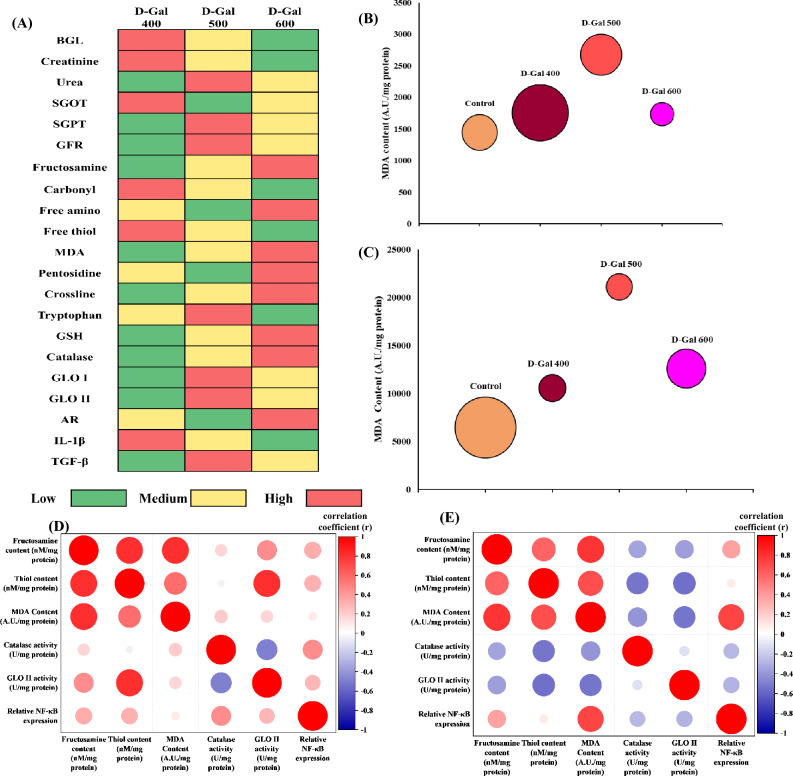
Correlation analysis of biochemical, glycation, oxidative stress, inflammatory, and detoxification parameters following chronic D-galactose (D-Gal) administration. Male Wistar rats were treated with subcutaneous injections of D-Gal (400, 500, and 600 mg/kg body weight) for 56 days. **A** Heatmap representation of biochemical, glycation, antioxidant, inflammatory, and detoxification parameters in D-Gal-treated groups. Color intensity indicates relative expression or activity levels of the analyzed markers, where green represents low activity, yellow represents moderate activity, and red represents high activity. Parameters analyzed include blood glucose level (BGL), creatinine, urea, SGOT, SGPT, glomerular filtration rate (GFR), fructosamine, carbonyl content, free amino groups, free thiol groups, malondialdehyde (MDA), pentosidine, crossline, tryptophan fluorescence, reduced glutathione (GSH), catalase activity, glyoxalase I (GLO I), glyoxalase II (GLO II), aldose reductase (AR), interleukin-1 beta (IL-1β), and transforming growth factor beta (TGF-β). **B** Bubble plot representing the association among MDA content, relative Nrf2 expression, and aldose reductase (AR) activity in liver tissue across control and D-Gal-treated groups. Bubble size indicates relative AR activity, whereas the vertical axis represents MDA levels, reflecting oxidative stress-associated alterations. **C** Bubble plot representing the association among MDA content, relative Nrf2 expression, and AR activity in kidney tissue. Increased MDA levels and altered AR activity demonstrate tissue-specific oxidative and glycation stress responses following D-Gal exposure. **D** Correlation matrix analysis of D-Gal 500-treated liver tissue showing relationships among fructosamine content, thiol content, MDA content, catalase activity, GLO II activity, and relative NF-κB expression. Positive correlations are represented by red-colored circles, whereas negative correlations are represented by blue-colored circles. Circle size and color intensity correspond to the degree of the correlation coefficient (r). **E** Correlation matrix analysis of D-Gal 500-treated kidney tissue demonstrating the interrelationship among glycation products, antioxidant enzyme activity, detoxification enzymes, lipid peroxidation markers, and inflammatory signaling pathways

As shown in Fig. 8A, the D-Gal 500 group showed a balanced effect on glycation, inflammation, and oxidative stress. Different markers, such as fructosamine, MDA, GSH, and catalase, were moderately preserved in the 500 group, suggesting partial maintenance of antioxidant defense. The relationship between aldose reductase activity (X-axis) and MDA levels (Y-axis), with bubble size indicating Nrf2 gene expression, was shown in a bubble plot. As observed in Fig. 8B and C, liver tissue showed a progressive increase in aldose reductase activity and MDA levels, accompanied by a decrease in Nrf2 expression, indicated by smaller bubble sizes. Among the three D-Gal doses, the 500 group showed the most significant effect in both liver and kidney tissue lysates. A correlation analysis was performed to provide an overall interpretation of the D-Gal 500 dose and its impact on glycation, oxidation, and inflammation. In liver tissue lysates, fructosamine content showed a strong positive correlation with MDA levels (r^2^ = 0.8), with no significant association observed with catalase or GLO II activity as shown in Fig. 8D. In kidney tissue lysates (Fig. 8E), fructosamine content also showed a positive correlation with MDA levels (r^2^ = 0.8) and relative NF-κB expression (r^2^ = 0.4), while exhibiting a negative correlation with antioxidant markers such as catalase and GLO II activity. Additionally, antioxidant enzymes demonstrated negative correlations, indicating a redox imbalance in liver and kidney tissues under D-Gal exposure at 400 and 600 mg doses (Supplementary Fig. 2).

## Discussion

Chronic D-Gal administration in the present study imbalanced glycation, oxidative stress, and inflammatory pathways, resulting in significant hepatic and renal dysfunction. Mechanistically, excess D-Gal promoted the formation of AGEs, which activated the RAGE-NF-κB signaling axis, while simultaneously impairing Nrf2-mediated antioxidant and detoxification responses. The reduction of D-Gal to galactitol diffuses across cell membranes, leading to an imbalance in ROS levels and dysregulation of biochemical, glycation, antioxidant, and detoxification activities. The D-Gal-induced toxicity impaired biochemical parameters, such as a depletion of albumin, creatinine, SGOT, SGPT, and BUN (Martinovic et al. 2025). D-Gal injection can lead to hepatocellular injuries along with elevated levels of several blood enzymes, such as SGOT and SGPT(Azman et al. 2021; Zhao et al. 2021; Zhang et al. 2024). Consistent with these findings, our study also observed a slight increase in urinary urea levels, which is indicative of impaired renal filtration. Elevated urinary urea concentration reflects a compensatory mechanism in response to glomerular dysfunction. Collectively, these biochemical alterations corroborate D-Gal's role in promoting progressive liver and kidney dysfunction.

Excess D-Gal promotes non-enzymatic glycation, leading to the formation and accumulation of advanced glycation end products (AGEs) (Shamsi et al. 2016). Elevated fructosamine levels at early time points reflect increased formation of early glycation products; however, the subsequent decline at later stages may indicate their conversion into AGEs (Song et al. 1999). This shift is consistent with the nature of glycation, in which early intermediates are transient and give rise to more stable AGEs over time. Glycation disrupts protein structure and function by modifying free amino and thiol groups (Goldin et al. 2006). A study by Song et al (1999) demonstrated that D-Gal treatment increases plasma AGE levels over time. Similarly, other studies have reported that D-Gal- or AGE-treated animals exhibit features comparable to naturally aged models (Samad et al. 2022; Zhang et al. 2023). In the present study, we showed that early glycation intermediates increased initially, followed by a marked elevation of AGE products, such as crossline, at later time points.

Antioxidant enzymes are the primary defense mechanisms against oxidative stress and inflammation. Among these, catalase plays a crucial role in decomposing hydrogen peroxide into water and oxygen and protects the cells from oxidative injury (Ighodaro and Akinloye 2018). Another vital antioxidant molecule, GSH, acts as a free radical scavenger and detoxifying agent that maintains cellular thiol balance (Carmo de Carvalho e Martins et al. 2022). However, under conditions of D-Gal-induced glycation and oxidative stress, GSH levels are significantly depleted, impairing the cellular antioxidant capacity (Umbayev et al. 2020; Saenno et al. 2025). Consistent with these reports, our study demonstrated a gradual, dose-dependent decline in both GSH content and catalase activity in D-Gal-treated groups. This downregulation reflects the intense oxidative burden induced by D-Gal exposure, thereby contributing to hepatic and renal dysfunction observed in this model.

The enzymes GLO I and II play a crucial role in neutralizing reactive carbonyl compounds such as methylglyoxal, which are elevated during glycation and oxidative stress (Arai et al. 2014). The brain senescence has been observed following D-Gal administration to mice, resulting in disturbed Glo I activity (Li et al. 2019). Our study revealed a dose-dependent depletion of GLO I and GLO II, showed an imbalance in the detoxification machinery. In parallel, AR, an enzyme involved in the polyol pathway, showed increased activity. While AR catalyzes the reduction of excess D-Gal into galactitol, this metabolite cannot be further degraded, leading to its intracellular accumulation and contributing to osmotic and oxidative stress (Azman et al. 2021). The observed overactivity of AR appears to be a compensatory defense mechanism aimed at mitigating ROS generation.

Accumulation of AGEs activates the RAGE-mediated signaling pathway and initiates NF-κB transcription factor activation in a positive feedback loop (Yano et al. 2020). Activated NF-κB translocates to the nucleus and elevates the production of pro-inflammatory cytokines, which sustains oxidative stress and inflammation. The report showed that D-Gal treatment significantly affects RAGE gene and protein expression. Further, it activates NF-κB and promotes inflammation, affecting conditions such as renal aging (Zeng et al. 2022). The same results were observed in the present study where the subcutaneous injection of D-Gal for 8 weeks increased the RAGE gene and protein levels and further activated the NF-κB and inflammatory cytokines. In addition to activation of the RAGE-NF-κB inflammatory pathway, D-Gal-induced oxidative stress critically disrupts the Nrf2-mediated antioxidant defense system. Under physiological conditions, Nrf2 regulates the expression of key antioxidant and detoxification enzymes. However, chronic D-Gal exposure leads to excessive ROS generation and impairs Nrf2 activation. This Nrf2 suppression reduces the expression of cytoprotective enzymes, further exacerbating oxidative damage. Importantly, a functional interplay exists between the NF-κB and Nrf2 pathways, in which NF-κB activation negatively regulates Nrf2 activity. In the present study, the upregulation of NF-κB and downregulation of Nrf2 indicate a shift toward a pro-inflammatory and pro-oxidant state. Thus, D-Gal-induced toxicity is mediated by the dysregulation of the RAGE-NF-κB-Nrf2 axis, which is triggered through glycation, oxidative stress, and inflammatory responses.

## Conclusion

The present study provides evidence that chronic D-Gal exposure induces a progressive accumulation of AGEs, oxidative stress, inflammation, and impaired detoxification across plasma, liver, and kidney tissues. These pathological alterations are characterized by significant upregulation of RAGE and NF-κB, suppression of Nrf2 and its downstream antioxidant genes (HO-1, NQO1), and notable histopathological damage in hepatic and renal tissues. Additionally, elevated levels of cytokines such as IL-1β and TGF-β1, along with downregulation of glyoxalase enzymes (GLO-I and GLO-II) and altered AR activity. Among all experimental groups, the D-Gal 500 consistently exhibited the most significant biochemical, molecular, and histological changes, confirming it as the optimal dose for modeling glyco-oxidative stress and age-associated tissue dysfunction.

## Supplementary Information

Below is the link to the electronic supplementary material.

Supplementary file1 (PPTX 1507 KB)

Supplementary file2 (DOCX 20 KB)

## Data Availability

The datasets generated and/or analysed in the current study are not publicly available due to ongoing research projects in our lab, but are available from the corresponding author upon reasonable request.
