# Supplementary material for: Modulation of glycation, inflammation, and detoxification pathways by D-Galactose through the RAGE–NF-κB–Nrf2 signaling axis in liver and kidney of Wistar rats
Source: 3 Biotech. 2026 Aug 2;16(8):364. doi: 10.1007/s13205-026-04988-5 (PMC13429561; doi:10.1007/s13205-026-04988-5)
Supplement: Supplementary file 1 — Supplementary file1 (PPTX 1507 KB) [file 13205_2026_4988_MOESM1_ESM.pptx]

## Slide 1
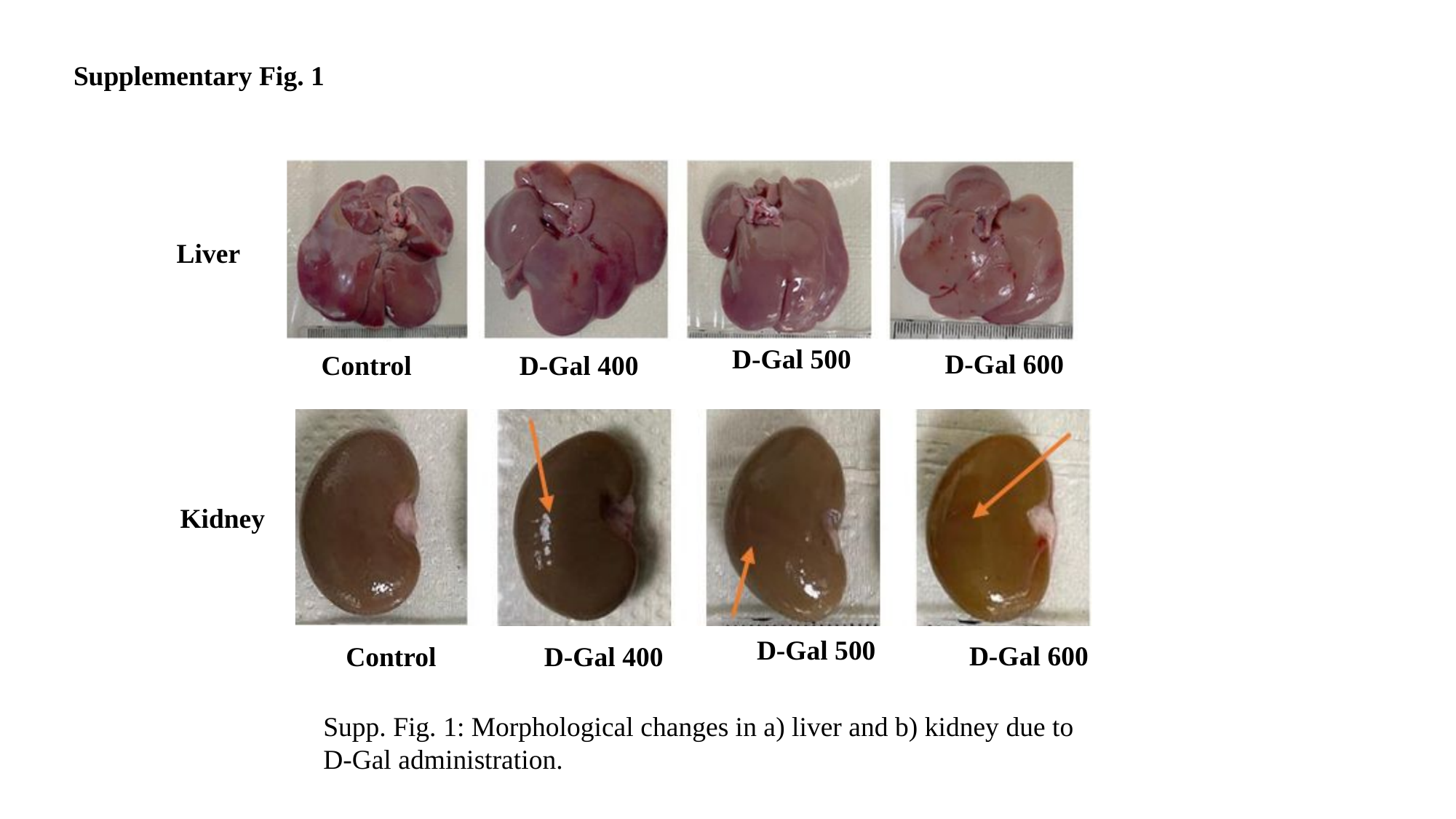

Supplementary Fig. 1
Liver
D-Gal 500
D-Gal 600
Control
D-Gal 400
Kidney
D-Gal 500
D-Gal 600
Control
D-Gal 400
Supp. Fig. 1: Morphological changes in a) liver and b) kidney due to D-Gal administration.

## Slide 2
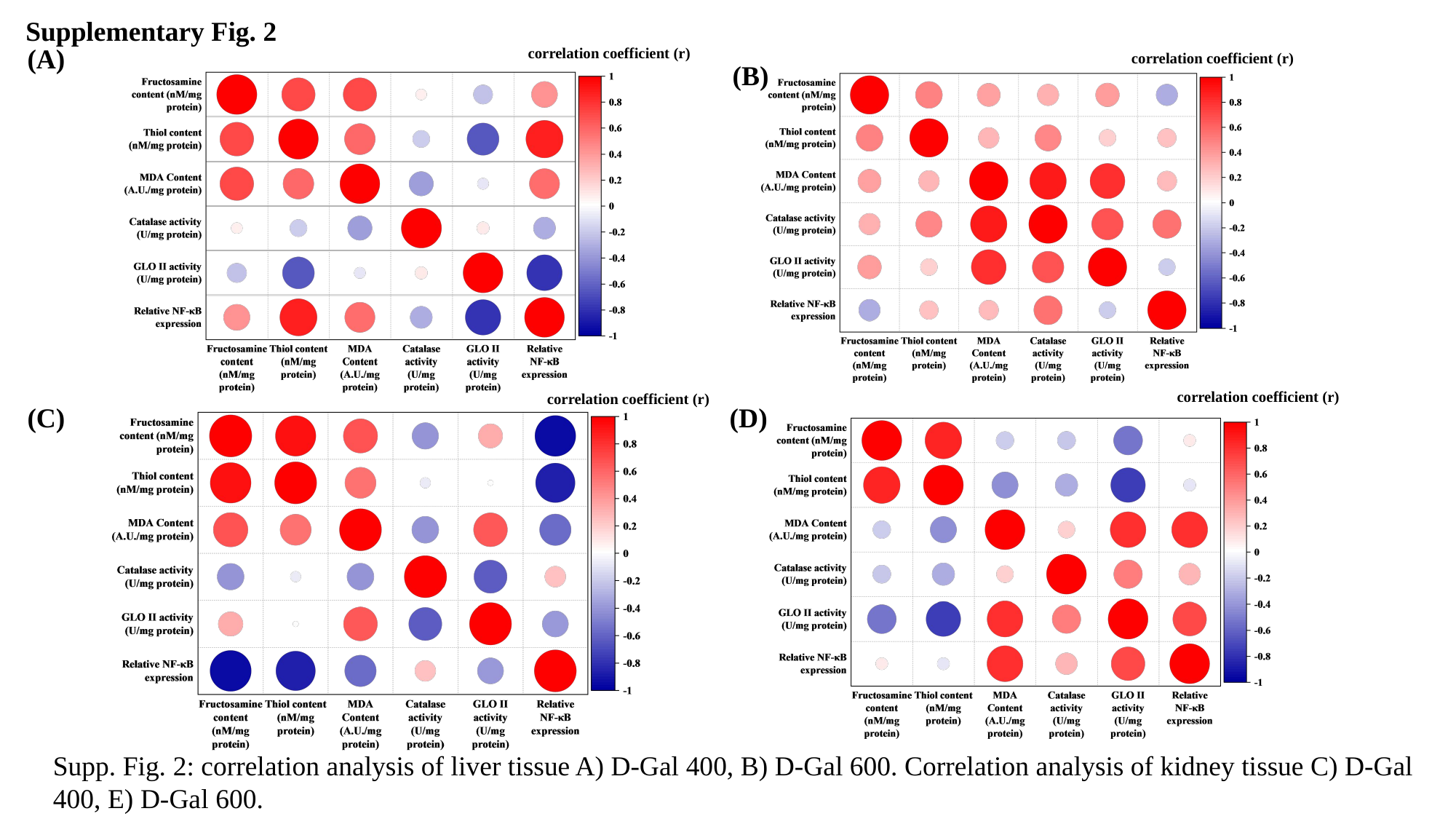

Supplementary Fig. 2
(A)
correlation coefficient (r)
correlation coefficient (r)
(B)
correlation coefficient (r)
correlation coefficient (r)
(C)
(D)
Supp. Fig. 2: correlation analysis of liver tissue A) D-Gal 400, B) D-Gal 600. Correlation analysis of kidney tissue C) D-Gal 400, E) D-Gal 600.
