# Supplementary material for: Modulation of glycation, inflammation, and detoxification pathways by D-Galactose through the RAGE–NF-κB–Nrf2 signaling axis in liver and kidney of Wistar rats
Source: 3 Biotech. 2026 Aug 2;16(8):364. doi: 10.1007/s13205-026-04988-5 (PMC13429561; doi:10.1007/s13205-026-04988-5)
Supplement: Supplementary file 2 — Supplementary file2 (DOCX 20 KB) [file 13205_2026_4988_MOESM2_ESM.docx]

Supplementary Table 1: Primers sequence used for qPCR analysis

| Sr No | Gene | Forward primer | Reverse primer |
| --- | --- | --- | --- |
| 1 | Nrf2 | 5ˈ-GCCTGGGTTCAGTGACTCGGA- 3 | 5ˈ-CTGTGCCCTTGAGCTGGCGA- 3ˈ |
| 2 | RAGE | 5ˈ-AGAAACCGGTGATGAAGGACA- 3ˈ | 5ˈ-GGTTGTCGTTTTCGCCACAG- 3ˈ |
| 3 | NF-κB | 5ˈ-AACAACACAGACCCAGGAGT- 3ˈ | 5ˈ-CTGTCACCAGGCGAGTTATAG- 3ˈ |
| 4 | NQO1 | 5ˈ- GACATCACAGGGGAGCCGA- 3ˈ | 5ˈ-AGCTACAATATCCGGGCTCAG- 3ˈ |
| 5 | HMOX1 | 5ˈ-CTGGTGATGGCCTCCTTGTA- 3ˈ | 5ˈ-GATGAGTACCTCCCACCTCG- 3 ˈ |
| 6 | GAPDH | 5ˈAGACAGCCGCATCTTCTTGT - 3ˈ | 5ˈ-CTTGCCGTGGGTAGAGTCAT - 3ˈ |
